# Editorial: Regulation of tumor-immune microenvironment by non-coding RNAs

**DOI:** 10.3389/fimmu.2024.1362881

**Published:** 2024-01-16

**Authors:** Rajeev Nema, Rohit Saluja, Ashok Kumar

**Affiliations:** ^1^ Department of Biosciences Manipal University Jaipur, Jaipur, Rajasthan, India; ^2^ Department of Biochemistry, All India Institute of Medical Sciences (AIIMS), Bibinagar, Telangana, India; ^3^ Department of Biochemistry, All India Institute of Medical Sciences (AIIMS), Bhopal, Madhya Pradesh, India

**Keywords:** tumor micro environment, noncoding RNA, long non coding RNA, exosomes, circular RNA (circRNA)

The tumor microenvironment (TME) is a vital component in carcinogenesis, invasion, progression, metastasis, and chemoresistance (1). Non-coding RNAs (NcRNAs) include small noncoding RNAs (sncRNAs) (< 50 bp in size) and long noncoding RNAs (lncRNAs) (> 200 bp in size). sncRNAs mainly include transfer RNAs, small interfering RNAs, microRNAs (miRNAs), PIWI-interacting RNAs (piRNAs), circular RNA (circRNA) small nuclear RNAs (snRNAs). Several ncRNAs are being tested as potential diagnostic, prognostic, and predictive biomarkers and also serve as potential therapeutic targets to improve immunotherapy efficacy (2). Immune evasion, a hallmark of cancer, promotes tumor growth hinders immunotherapy. Immune checkpoint inhibitors have shown potential for increasing patient survival outcomes, but a significant fraction of patients remain unresponsive due to the immunosuppressive tumor environment(3). The effective antitumor immune response depends on the activation and interaction of various tumor-infiltrating lymphocytes (TILs), such as T cells, B cells, and natural killer cells (NK cells). These cells exert both positive and negative regulatory functions in the antitumor immunity process. Furthermore, depending on the context and stimuli, CD4+ helper T cells (Th cells) differentiate into different subsets including, Th1, Th2, Th9 Th17 cells, and regulatory T cells (Tregs). These T cell subsets secrete different cytokines and chemokines and thereby orchestrate the TME and affect the prognosis of cancer patients (**Figure 1**). This Research Topic is focused on the regulatory and prognostic roles of lncRNAs and circRNA on T cell differentiation, exhaustion, and their potential as therapeutic targets for cancer immunotherapy.

**Figure 1:**
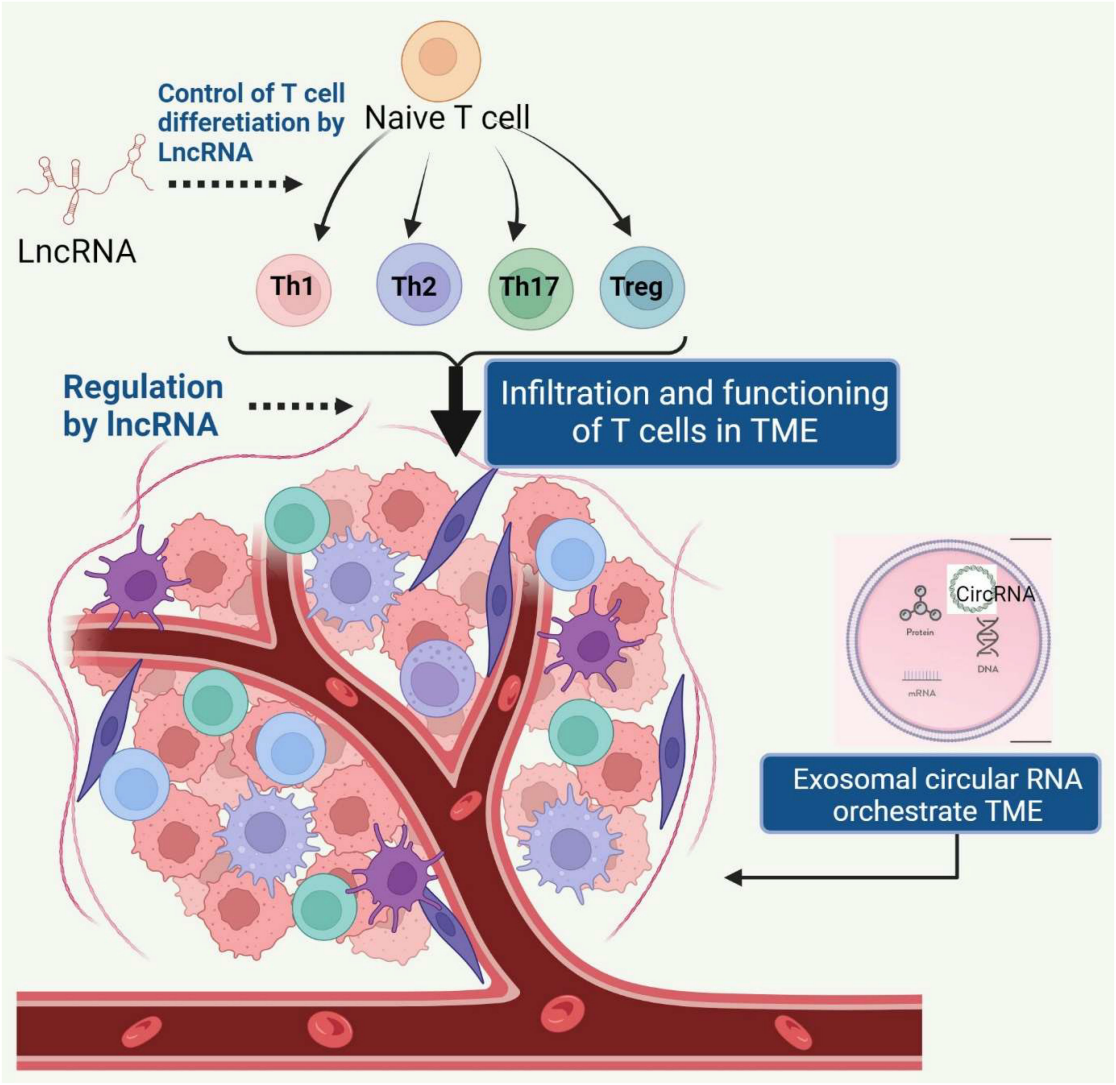
Various LncRNAs regulate the differentiation of naïve T cells into different subsets under homeostasis and during carcinogenesis. These subsets exhibit unique landscape of lncRNAs, which also control the trafficking of T cell subsets into tumor microenvironment (TME). Exosomal circular RNAs released by cancer cells and stromal cells orchestrate the TME.

In this Research Topic, Erber and Herndler-Brandstetter have put forth a comprehensive review on the regulatory role of lncRNA in T cell differentiation under homeostasis and cancer. Authors have reviewed that different T cell subsets express lineage specific lncRNAs and Th1-, Th2- and Th17 subsets have unique signature of lncRNAs. Similarly, naïve, effector and memory CD8+ T cells express unique lncRNAs. Studies have demonstrated that CD4+ and CD8+ T cells can be distinguished based on their lncRNA expression profile. However, limited information is available on the expression and role of lncRNAs in tumor-infiltrating T cell subsets and their impact on T cell function, tumor immune escape, and the efficacy of T cell-based cancer immunotherapies.

Gastric cancer (GC) ranking fourth in terms of incidence and fifth in morbidity among all types of cancers. Several lncRNAs have the potential to influence chemoresistance by modulating genes associated with DNA damage repair (DDR). Zhao et al constructed a prognostic model for GC by analyzing The Cancer Genome Atlas (TCGA) and Gene Expression Omnibus (GEO) datasets. Based on the expression profile of five lncRNAs (AC007405.3, AC145285.6, AL590705.3, LINC00106 and MAGI2-AS3), the model classifies patients into low- and high-risk groups. Patients with a low-risk score had better overall survival and prognosis. The prognostic model plays a significant role in tumor metastasis, clinicopathological characteristics, prognosis, microsatellite instability, and drug sensitivity. Further, authors demonstrated that the GC patients treated with immunotherapy in the low-RS score group had longer survival and better prognosis than those in the high-risk score group. The study gives insight into improving the prognosis for patients with GC and broadening our understanding on lncRNAs related to DDR.

Renal cell carcinoma (RCC) is a prevalent malignant tumor affecting the urinary system. Clear cell RCC (ccRCC), a major subtype of RCC, exhibits susceptibility to immune infiltration, and the attributes of the TME significantly impact the immunotherapy response. Deng et al. have identified and validated 10 TILs-related lncRNAs in ccRCC. Authors constructed a risk-score model based on TILs-related-lncRNAs, and a nomogram to predict the overall survival of ccRCC patients. Authors furthers demonstrated that TIL-related lncRNA signature panel was associated with T cell function. Notably, authors showed that one of the lncRNA, AC084876.1-associated with the activation and differentiation of tumor infiltrating Tregs, may serve as a potential therapeutic target for ccRCC. Colorectal cancer (CRC) is a prevalent malignancy affecting the digestive system. Cancer-associated fibroblasts (CAFs), integral components of the TME, secrete various substances, including exosomes, growth factors and cytokines. Exosome-derived ncRNAs play a crucial role in CRC. Sun et al. have reviewed the role of CAFs-derived exosomal ncRNAs in regulating the tumor microenvironment and influencing the growth of CRC in metastasis. Authors have further emphasized that CAFs-derived exosomes can be used as biomarkers for companion diagnosis of CRC metastasis, drug resistance and prognosis. Hepatocellular carcinoma (HCC), the predominant primary malignancy affecting the liver. Exosomal circRNAs, secreted by HCC cells, stimulate angiogenesis, contribute to metabolic reprogramming, facilitate inflammatory changes, and induce tumor immunosuppression. The exosomes secreted by HCC cells transport circRNA into immune cells and promote the overexpression of immune checkpoints to regulate immune response, leading tumor cells to acquire immunosuppressive properties. With advancements in detection methods, experimental methodologies, and bioinformatics algorithms, exosomal circRNAs are expected to play a crucial role in tumor diagnosis and treatment. Xu et al. have reviewed the role of exosomal circRNAs in shaping the TME of HCC and controlling the interaction of exosomes and TILs.

Plant-derived RNAs, particularly small RNAs, has shown promising results in anti-tumor performance, immune regulation, and antiviral activities in macrophage-mediated anti-tumor/inflammatory therapies. These plant RNAs have shown cross-kingdom regulation by functional mRNAs and miRNAs, their involvement and potentials in macrophage-mediated anti-tumor/inflammatory therapies, and their load prospects in viruses and natural exosome vehicles. Medicinal plant nucleic acids have demonstrated advantages such as large gene banks, easy availability, and low toxicity, making them a potential treasure house for gene therapy. Liu et al, has emphasized on plant-derived RNAs as next generation drugs with potential application in nucleic acid-based biotherapy. Authors also discuss the challenges and bottlenecks for the development of plant-derived nucleic acids into biodrugs for human diseases therapy.

Several lncRNAs serve as prognostic markers for various cancer types including cervical cancer, gastric cancer and hepatocellular cancer. Large-scale randomized clinical trials remain a major gap in ncRNA-targeted therapeutics, necessitating more original studies to understand their clinical value and impact on cancer progression.

## Author contributions

RN: Writing – original draft, Writing – review & editing. RS: Writing – review & editing. AK: Conceptualization, Supervision, Writing – original draft, Writing – review & editing.
